# Supplementary material for: A Likelihood Approach for Real-Time Calibration of Stochastic Compartmental Epidemic Models
Source: PLoS Comput Biol. 2017 Jan 17;13(1):e1005257. doi: 10.1371/journal.pcbi.1005257 (PMC5240920; doi:10.1371/journal.pcbi.1005257)
Supplement: S1 File — (TAR.GZ) [file pcbi.1005257.s014.tar.gz › HSPH_Online-SI-Revision/EnKF07/n100-mild/n100-mild_EnKF_table4.pdf]

1	R0	{1.24125, 3.38982}	{2.27895, 9.44984}	{{0.492878, 6.24343}, {3., 19.2528}}	{112.408, 178.771}	inf	918.	7838.36	{303.025, 10 201.}	774.452	0.726114	newly	1628.	6732.95	{157., 7924.}	349.302	0.529467	Reff	0.854936	0.612118	{0.273148, 0.936466}	40.2769	ar	34 926.	180 824.	{165 134., 191 038.}	417.736
2	R0	{1.29554, 4.51878}	{2.33851, 3.05051}	{{0.0747973, 1.5692}, {3., 3.87198}}	{116.274, 32.4926}	inf	1374.	9708.06	{1832.26, 28 261.3}	610.922	0.651384	newly	1875.	14 556.6	{1854., 26 965.}	683.346	0.670143	Reff	0.799646	0.555569	{0.0515981, 1.01824}	53.4559	ar	42 828.	184 823.	{162 869., 208 010.}	331.547
3	R0	{1.2443, 3.64292}	{2.54409, 1.8371}	{{1.6161, 0.414758}, {3., 3.29943}}	{104.644, 49.5706}	inf	748.	1801.42	{174.638, 2272.42}	181.631	0.475342	newly	1275.	8137.9	{386., 5624.}	575.349	0.524234	Reff	0.825668	0.672173	{0.275875, 1.03385}	41.8024	ar	37 087.	173 236.	{162 318., 184 889.}	367.106
4	R0	{1.38465, 3.43331}	{2.68366, 4.46712}	{{2.18443, 0.167279}, {3., 5.80939}}	{93.8151, 62.2082}	inf	741.	4541.64	{1884.12, 8234.32}	513.509	0.581164	newly	1149.	7242.2	{1713., 17 531.}	531.939	0.477725	Reff	0.722441	0.593867	{0.247454, 1.427}	57.6993	ar	49 511.	186 974.	{167 495., 250 155.}	277.64
5	R0	{1.25409, 4.1068}	{2.53974, 1.85222}	{{1.65594, 0.431083}, {3., 3.31323}}	{102.888, 54.8986}	inf	1115.	1526.13	{292.645, 2269.22}	87.5198	0.332812	newly	1641.	6528.82	{428., 4251.}	339.264	0.368112	Reff	0.822708	0.603394	{0.0808929, 1.12853}	48.0308	ar	38 571.	171 922.	{161 400., 181 524.}	345.729
6	R0	{1.28821, 4.2016}	{2.83306, 3.24067}	{{2.44353, 0.14905}, {3., 6.47135}}	{119.922, 75.3736}	inf	1013.	1651.03	{234.524, 1680.29}	140.297	0.371928	newly	1399.	2405.59	{467., 3723.}	131.66	0.338413	Reff	0.800326	0.718922	{0.242235, 1.10884}	49.5083	ar	41 468.	168 202.	{155 653., 233 716.}	305.619
7	R0	{1.24369, 2.56007}	{2.53969, 5.84504}	{{1.71869, 3.01596}, {3., 7.70608}}	{104.206, 128.316}	inf	442.	3726.96	{300.302, 4141.83}	757.733	0.675524	newly	924.	5163.58	{224., 5806.}	490.929	0.495439	Reff	0.781634	0.487065	{0.244268, 0.784695}	47.613	ar	38 603.	171 123.	{160 520., 176 537.}	343.29
8	R0	{1.232, 3.53942}	{2.53675, 3.8471}	{{2.12823, 3.41812}, {3., 4.32086}}	{121.351, 10.8317}	inf	649.	8228.39	{1281.74, 12 893.3}	1180.01	0.936218	newly	1057.	13 429.9	{1516., 43 643.}	1184.17	0.907325	Reff	0.831846	0.759786	{0.0250382, 2.1282}	76.8005	ar	34 555.	190 234.	{161 638., 250 806.}	450.526
9	R0	{1.36262, 3.85631}	{2.10107, 7.62187}	{{0.802075, 5.79212}, {3., 8.59659}}	{83.1324, 97.6468}	inf	951.	4698.59	{630.177, 2912.97}	411.095	0.410874	newly	1368.	3330.4	{399., 2395.}	188.883	0.295705	Reff	0.74132	0.381932	{0.152371, 0.567641}	49.1682	ar	48 410.	171 573.	{158 440., 187 009.}	254.417
10	R0	{1.28702, 4.67968}	{2.65307, 3.2383}	{{1.83428, 1.34286}, {3., 4.86002}}	{106.141, 32.6429}	inf	1183.	8827.16	{456.097, 38 747.9}	666.295	0.667121	newly	1557.	16 979.8	{603., 71 990.}	1010.74	0.768743	Reff	0.80682	0.739278	{0.123269, 1.08438}	46.4667	ar	41 092.	195 495.	{162 490., 284 064.}	375.748
11	R0	{1.35752, 3.47129}	{2.52563, 1.8894}	{{1.76663, 0.281947}, {3., 3.29613}}	{86.4467, 45.5705}	inf	1161.	1932.58	{652.812, 2990.78}	85.9857	0.252258	newly	1963.	8523.83	{798., 5960.}	354.143	0.359978	Reff	0.799932	0.682952	{0.35412, 0.923377}	39.8416	ar	44 669.	174 066.	{164 516., 177 592.}	289.68
12	R0	{1.35929, 5.83647}	{2.49126, 3.37332}	{{1.54546, 2.86318}, {3., 5.04878}}	{83.2766, 42.2028}	inf	1940.	11 919.3	{2410.27, 46 536.}	521.195	0.599007	newly	2036.	17 321.8	{4329., 57 598.}	757.007	0.702503	Reff	0.813535	0.632398	{0.261971, 1.10563}	39.7006	ar	46 715.	197 934.	{169 211., 282 252.}	323.705
13	R0	{1.28019, 3.29908}	{2.70316, 9.84613}	{{2.28573, 4.52921}, {3., 13.6306}}	{111.179, 198.451}	inf	878.	2866.5	{488.076, 5958.24}	244.311	0.424087	newly	1478.	2582.07	{212., 7472.}	140.861	0.475397	Reff	0.791618	0.689938	{0.161879, 2.25604}	70.197	ar	40 941.	177 102.	{158 978., 245 678.}	332.58
14	R0	{1.36661, 3.48318}	{2.42682, 5.53374}	{{1.59629, 3.09474}, {3., 7.46921}}	{77.5795, 66.2496}	inf	608.	5106.81	{251.536, 10 172.1}	770.057	0.660183	newly	929.	7454.04	{219., 16 118.}	743.744	0.617178	Reff	0.726053	0.532934	{0.1493, 0.925535}	43.2092	ar	48 672.	177 247.	{160 603., 204 952.}	264.166
15	R0	{1.35865, 3.84123}	{2.30877, 1.91206}	{{0.768215, 0.150573}, {3., 3.37844}}	{89.1325, 50.2228}	inf	1150.	2016.53	{707.535, 3168.17}	91.6023	0.268068	newly	1651.	8972.25	{1158., 5233.}	455.534	0.369359	Reff	0.73825	0.611709	{0.110482, 1.61838}	52.7101	ar	48 598.	173 080.	{161 251., 218 691.}	256.147
16	R0	{1.22879, 3.98329}	{2.49421, 3.10359}	{{1.59473, 2.20696}, {3., 4.63628}}	{102.981, 28.9661}	inf	1035.	13 894.3	{7053.92, 42 593.1}	1243.61	0.971297	newly	1786.	20 759.3	{6616., 70 606.}	1064.23	0.853769	Reff	0.876398	0.552701	{0.223423, 1.00282}	47.725	ar	34 405.	206 217.	{176 438., 285 639.}	499.382
17	R0	{1.34925, 3.64524}	{2.25165, 7.55974}	{{0.810202, 3.91411}, {3., 8.86455}}	{89.9277, 107.387}	inf	1239.	4689.66	{390.2, 2247.27}	301.915	0.403825	newly	1851.	3414.29	{385., 1897.}	146.338	0.341976	Reff	0.759911	0.389898	{0.114239, 0.693541}	55.531	ar	46 871.	171 556.	{157 774., 173 900.}	266.017
18	R0	{1.33054, 2.25275}	{2.02803, 5.79804}	{{0.742481, 3.67579}, {3., 11.6372}}	{85.147, 157.376}	inf	312.	5181.65	{288.113, 4180.49}	1572.92	0.906561	newly	665.	5147.64	{312., 5465.}	699.248	0.640231	Reff	0.750025	0.389779	{0.129898, 0.596176}	50.9475	ar	44 336.	174 094.	{163 235., 172 758.}	292.671
19	R0	{1.28394, 3.69859}	{2.72791, 3.14452}	{{2.1223, 2.08718}, {3., 3.93914}}	{112.464, 19.013}	inf	963.	7650.82	{1386.34, 14 629.1}	694.822	0.673047	newly	1523.	18 622.6	{1806., 62 730.}	1126.24	0.741465	Reff	0.824444	0.897713	{0.234125, 2.12228}	59.1647	ar	39 558.	196 968.	{164 470., 273 890.}	397.921
20	R0	{1.2883, 2.26493}	{1.93506, 5.67755}	{{0.774966, 3.66804}, {3., 11.6219}}	{84.285, 152.643}	inf	531.	8117.61	{605.216, 6173.83}	1436.8	0.795103	newly	1246.	7928.39	{626., 6975.}	562.06	0.520979	Reff	0.78634	0.366602	{0.104419, 0.605846}	53.4808	ar	40 768.	178 978.	{161 503., 186 443.}	339.015
21	R0	{1.34098, 2.78621}	{1.83278, 4.49376}	{{0.767605, 2.60439}, {3., 11.7393}}	{76.1374, 65.6186}	inf	549.	6033.02	{273.552, 4175.27}	1017.19	0.792078	newly	1041.	7025.05	{384., 5993.}	606.091	0.634763	Reff	0.763192	0.357396	{0.137501, 0.546109}	55.2684	ar	44 605.	177 477.	{164 540., 175 464.}	297.886
22	R0	{1.33783, 5.95911}	{2.64446, 3.00059}	{{1.7863, 0.868941}, {3., 4.78131}}	{97.6675, 49.6469}	inf	1990.	8128.62	{1502.93, 36 830.8}	316.594	0.400111	newly	2166.	15 194.8	{1369., 67 430.}	611.304	0.565643	Reff	0.826485	0.678559	{0.0787887, 1.24106}	49.6353	ar	45 418.	187 460.	{158 399., 289 821.}	312.743
23	R0	{1.33359, 3.3069}	{2.64822, 10.8718}	{{1.67903, 5.44812}, {3., 15.3353}}	{99.3047, 228.762}	inf	1009.	1915.63	{975.531, 1708.64}	98.8211	0.231052	newly	1653.	1440.37	{389., 3286.}	78.3829	0.386532	Reff	0.751291	0.590521	{0.225313, 2.27369}	68.5968	ar	46 245.	172 977.	{159 441., 246 139.}	274.044
24	R0	{1.30588, 2.56123}	{2.47104, 5.81178}	{{2.00589, 1.32562}, {3., 17.3136}}	{94.2188, 170.278}	inf	372.	6950.97	{471.969, 9047.32}	1777.97	0.969292	newly	702.	12 713.4	{514., 29 292.}	1724.42	0.792291	Reff	0.762347	0.636433	{0.212632, 2.00587}	68.1938	ar	42 383.	187 868.	{160 047., 257 243.}	343.262
25	R0	{1.29813, 4.51849}	{2.50524, 3.03261}	{{1.5442, 0.698083}, {3., 4.8489}}	{92.9882, 36.2418}	inf	1528.	13 933.5	{3744.66, 46 143.9}	818.636	0.788052	newly	2037.	16 755.7	{6398., 50 444.}	728.326	0.753641	Reff	0.813343	0.488043	{0.181101, 0.763453}	49.6014	ar	43 371.	203 967.	{172 525., 276 479.}	370.283
26	R0	{1.33231, 3.99953}	{2.17004, 10.2309}	{{0.741288, 6.20544}, {3., 19.1219}}	{94.8394, 155.802}	inf	1265.	7384.05	{561.667, 10 535.9}	502.792	0.551486	newly	1843.	6056.58	{253., 9745.}	269.682	0.468485	Reff	0.789434	0.84456	{0.253086, 1.16873}	42.7907	ar	44 733.	184 682.	{161 757., 206 707.}	312.854
27	R0	{1.30555, 4.61445}	{2.52667, 2.95871}	{{1.56532, 1.10971}, {3., 4.8163}}	{93.5326, 37.8083}	inf	1370.	12 950.5	{6283.57, 45 110.5}	847.943	0.813855	newly	1833.	17 257.1	{6218., 53 830.}	842.825	0.777054	Reff	0.814154	0.469595	{0.170345, 0.625644}	48.2594	ar	42 624.	198 055.	{169 541., 274 585.}	364.656
28	R0	{1.26819, 5.34969}	{2.54631, 3.21966}	{{2.1604, 2.33561}, {3., 3.89837}}	{113.326, 39.816}	inf	1478.	5889.38	{1129.91, 6376.91}	310.319	0.435081	newly	1792.	11 692.	{2265., 27 582.}	560.153	0.585066	Reff	0.858552	0.83687	{0.124638, 2.16038}	61.0187	ar	38 946.	186 731.	{160 921., 248 094.}	379.462
29	R0	{1.19925, 3.00394}	{2.37955, 3.2005}	{{1.18361, 1.72821}, {3., 4.73859}}	{105.798, 49.8613}	inf	570.	1335.72	{460.829, 1183.18}	148.238	0.195101	newly	1093.	2011.52	{364., 1784.}	136.104	0.297786	Reff	0.866182	0.518786	{0.0849256, 0.829629}	47.1685	ar	31 727.	168 137.	{158 545., 200 551.}	429.948
30	R0	{1.35749, 3.75483}	{2.58109, 7.64544}	{{1.24371, 3.86392}, {3., 9.96383}}	{94.6615, 115.231}	inf	1015.	3266.82	{322.089, 4362.07}	259.54	0.382424	newly	1413.	4855.77	{256., 5357.}	318.413	0.474784	Reff	0.732529	0.531287	{0.121543, 0.656216}	65.0153	ar	48 244.	171 427.	{156 167., 242 406.}	255.334
31	R0	{1.27131, 4.2161}	{2.17044, 9.61307}	{{0.571331, 6.3921}, {3., 19.3205}}	{103.326, 128.009}	inf	1215.	5877.01	{247.328, 10 251.9}	416.068	0.595761	newly	1803.	5373.26	{126., 8501.}	252.786	0.590183	Reff	0.843883	0.675456	{0.138229, 1.13876}	41.3827	ar	38 299.	176 317.	{159 429., 197 167.}	360.37
32	R0	{1.31827, 6.18119}	{2.56632, 3.28173}	{{1.73228, 1.85108}, {3., 4.97165}}	{94.6735, 46.9078}	inf	1873.	10 795.	{2923.47, 44 735.8}	486.975	0.573242	newly	1902.	17 556.	{4144., 66 914.}	829.843	0.69697	Reff	0.818804	0.702519	{0.270496, 1.07601}	42.5414	ar	45 262.	199 978.	{164 525., 285 645.}	341.824
33	R0	{1.20398, 1.35167}	{2.16449, 6.20609}	{{0.795699, 3.65593}, {3., 11.8851}}	{101.723, 359.142}	inf	90.	4487.75	{135.228, 3846.25}	4894.02	1.3453	newly	298.	4326.99	{163., 5082.}	1370.41	0.873088	Reff	0.806895	0.425274	{0.124337, 0.620497}	53.5221	ar	33 329.	173 229.	{162 848., 171 415.}	419.754
34	R0	{1.32776, 3.146}	{2.10269, 7.47072}	{{0.820741, 5.79493}, {3., 8.60183}}	{85.7291, 137.467}	inf	466.	4575.62	{532.033, 5757.48}	885.679	0.648908	newly	799.	3272.22	{450., 4351.}	327.9	0.371444	Reff	0.745949	0.406367	{0.102, 0.712789}	47.1338	ar	44 872.	171 884.	{158 239., 186 143.}	283.055
35	R0	{1.279, 1.11336}	{1.99744, 2.69679}	{{0.734522, 0.670662}, {3., 3.66853}}	{89.9387, 178.433}	inf	15.	4120.5	{659.824, 3654.51}	27 372.2	2.0742	newly	47.	6454.69	{693., 6545.}	13 636.1	1.6792	Reff	0.754597	0.406901	{0.107641, 0.725686}	55.5204	ar	41 033.	164 065.	{154 460., 164 754.}	299.836
36	R0	{1.20029, 1.94717}	{1.91399, 5.82853}	{{0.884899, 3.61664}, {3., 11.7697}}	{82.8174, 199.334}	inf	338.	6638.11	{141.072, 4429.82}	1881.85	0.966118	newly	910.	6370.64	{174., 5872.}	634.675	0.668935	Reff	0.82688	0.414909	{0.138237, 0.702132}	52.4299	ar	33 234.	178 359.	{163 911., 190 290.}	436.676
37	R0	{1.33216, 3.69685}	{2.15096, 3.1997}	{{0.814027, 1.72629}, {3., 4.93637}}	{81.1809, 44.1203}	inf	945.	1250.85	{420.464, 2086.46}	85.7876	0.249563	newly	1507.	1670.4	{348., 2136.}	72.6821	0.278788	Reff	0.770335	0.449637	{0.130226, 0.701343}	43.2822	ar	45 235.	164 535.	{159 263., 169 413.}	263.735
38	R0	{1.25434, 2.04314}	{2.41178, 6.05138}	{{1.38531, 3.50589}, {3., 11.4259}}	{92.275, 196.18}	inf	246.	7072.94	{1444.58, 4546.36}	2775.69	1.23101	newly	612.	5656.33	{1888., 9664.}	826.446	0.805986	Reff	0.777829	0.457364	{0.0802405, 0.707733}	58.276	ar	38 662.	181 266.	{166 231., 221 454.}	368.849
39	R0	{1.27466, 3.0741}	{2.49172, 5.81351}	{{1.72486, 3.22425}, {3., 7.73492}}	{95.4808, 89.1127}	inf	499.	4437.79	{356.971, 5246.97}	805.258	0.584019	newly	937.	5588.42	{225., 8208.}	542.766	0.542821	Reff	0.783495	0.437155	{0.0940045, 0.756689}	51.5805	ar	40 181.	174 597.	{160 032., 183 795.}	334.525
40	R0	{1.33045, 2.68154}	{2.36672, 5.95339}	{{1.00853, 1.34454}, {3., 17.5971}}	{87.32, 169.189}	inf	318.	7524.9	{444.375, 20 931.8}	2271.93	1.04825	newly	616.	11 406.4	{614., 25 053.}	1760.42	0.860059	Reff	0.729978	0.63135	{0.221932, 1.49541}	57.8687	ar	45 951.	183 235.	{156 593., 253 483.}	298.761
41	R0	{1.37003, 2.83984}	{1.92743, 4.93649}	{{0.737626, 2.7859}, {3., 11.7595}}	{78.8563, 74.6399}	inf	516.	4792.13	{427.271, 4432.99}	841.315	0.720401	newly	894.	5138.01	{461., 5603.}	493.867	0.548989	Reff	0.718704	0.36775	{0.130888, 0.551028}	50.1176	ar	48 926.	174 357.	{162 736., 181 085.}	256.369
42	R0	{1.3003, 2.94885}	{2.53773, 5.74543}	{{1.71857, 3.11991}, {3., 7.69724}}	{95.1653, 94.8363}	inf	576.	3544.7	{127.759, 5768.22}	540.59	0.64006	newly	1090.	5581.72	{91., 7374.}	456.106	0.560735	Reff	0.746177	0.544953	{0.189198, 0.967918}	47.2417	ar	44 237.	173 709.	{159 474., 186 215.}	292.679
43	R0	{1.33756, 2.92653}	{1.95579, 5.71683}	{{0.73074, 3.68395}, {3., 11.4546}}	{83.556, 95.3449}	inf	776.	7606.61	{248.669, 8205.02}	901.761	0.720974	newly	1372.	7411.71	{232., 5611.}	474.907	0.574847	Reff	0.730161	0.380657	{0.141035, 0.611943}	48.2508	ar	47 342.	177 758.	{161 235., 191 700.}	275.477
44	R0	{1.35801, 2.92276}	{2.0351, 6.7094}	{{0.312172, 1.08887}, {3., 17.133}}	{104.908, 182.757}	inf	436.	6284.82	{1806.94, 5337.62}	1343.38	1.01401	newly	782.	7959.49	{651., 10 165.}	929.569	0.793161	Reff	0.722081	0.363963	{0.0513453, 0.562692}	57.5659	ar	47 940.	178 674.	{166 816., 204 122.}	272.704
45	R0	{1.31342, 4.02649}	{2.72197, 10.8089}	{{2.28538, 5.89177}, {3., 16.2477}}	{107.303, 168.444}	inf	1233.	1474.47	{1039.07, 1774.44}	44.1214	0.109233	newly	1772.	979.61	{384., 1039.}	66.7602	0.418893	Reff	0.7963	0.545306	{0.253743, 0.822841}	57.5496	ar	42 995.	169 799.	{159 606., 193 233.}	294.928
46	R0	{1.37919, 3.75889}	{2.28395, 7.59298}	{{0.808377, 5.71305}, {3., 8.41913}}	{88.7321, 102.001}	inf	1312.	5880.19	{484.796, 14 300.9}	365.197	0.447254	newly	1998.	4542.39	{297., 13 255.}	172.811	0.338284	Reff	0.747438	0.40727	{0.084428, 0.652204}	61.5612	ar	49 157.	176 711.	{157 747., 225 771.}	259.483
47	R0	{1.37458, 3.56117}	{2.14539, 7.3375}	{{0.805901, 5.61531}, {3., 8.18661}}	{83.815, 106.042}	inf	838.	4540.69	{1508.41, 5040.17}	446.595	0.494497	newly	1272.	3460.41	{991., 3664.}	190.215	0.249385	Reff	0.732981	0.34856	{0.0753788, 0.57937}	59.2312	ar	48 478.	173 350.	{159 455., 215 219.}	257.584
48	R0	{1.33393, 3.78296}	{2.66571, 3.11463}	{{2.09651, 1.77799}, {3., 3.96674}}	{99.8387, 20.8731}	inf	1093.	8040.08	{1367.14, 19 167.7}	642.917	0.702064	newly	1686.	19 575.6	{1290., 69 124.}	1073.19	0.812935	Reff	0.779095	0.936548	{0.252754, 2.09649}	61.2408	ar	44 885.	195 859.	{162 989., 267 630.}	336.357
49	R0	{1.24918, 2.39046}	{2.46886, 5.72152}	{{1.71334, 3.01728}, {3., 7.72553}}	{97.6384, 139.348}	inf	342.	4747.92	{174.508, 5257.07}	1304.05	0.810185	newly	806.	6513.71	{153., 8500.}	739.396	0.606883	Reff	0.817189	0.48301	{0.114231, 0.85427}	49.8063	ar	35 969.	174 926.	{162 729., 192 887.}	386.324
50	R0	{1.28281, 2.3032}	{2.69299, 4.39156}	{{2.18542, 0.22367}, {3., 5.92655}}	{109.929, 121.235}	inf	333.	4105.48	{1731.97, 8659.63}	1138.5	0.908963	newly	744.	6346.92	{1522., 12 151.}	758.513	0.637043	Reff	0.782989	0.616483	{0.211261, 1.2064}	55.5595	ar	40 223.	181 017.	{163 382., 212 423.}	350.033
